# Draft genome sequence of a black yeast fungus *Exophiala xenobiotica* isolated from La Brea Tar Pits

**DOI:** 10.1128/mra.00913-23

**Published:** 2024-01-05

**Authors:** Tania Kurbessoian, Danielle Stevenson, Renata Haro, Samantha Ying, Jason E. Stajich

**Affiliations:** 1 Department of Microbiology and Plant Pathology, Institute for Integrative Genome Biology, University of California, Riverside, California, USA; 2 Department of Environmental Studies, University of California, Riverside, California, USA; University of Strathclyde, Glasgow, Scotland, United Kingdom

**Keywords:** La Brea Tar Pits, extremophilic, black yeast, exophiala

## Abstract

A 30.28 Mb draft genome sequence was assembled and annotated for the melanized ascomycetous fungus *Exophiala xenobiotica* NRRL_64630 (Pezizomycotina; Chaetothyriales) isolated from La Brea Tar Pits, Los Angeles, California. Species identification was made by phylogenetic assessment of the Internal Transcribed Spacer. This is the first isolated fungal species from this historic space.

## ANNOUNCEMENT

Popular references to tar pits entertain vivid images of large mastodons and saber-tooth cats ensnared in a sticky substance that oozes from the ground (1). Asphalt and tar are similar hydrocarbon substrates, asphalt is released into seeps and collected in large viscous pools while tar is man-made (2). Seeps are found in many parts of the world, from Peru’s Talara Tar Pits to the La Brea Tar Pits (LBTP) (3). Culturally, the indigenous Los Angeles Native Gabrieleño-Tongva used tar as an adhesive on their boats and tools as far back as 10,000 years ago (3).

The role microbes and fungi can play in the remediation of tar-soaked soils remains an important opportunity for new technology. Fossils were collected from the LBTP in the early 1900s by UC Berkeley, LA High School, and the Southern California Academy of Sciences eventually leading to LBTP in the 1970s (4).

First, 5 mm of soil was collected from the La Brea site “Project 23” neighboring the Los Angeles County Museum of Art parking structure Box 5B (now found within the museum grounds). Cultures of fungi were obtained by plating dilutions of soils on nutrient-rich Malt Extract Yeast Extract (MEYE) media. Culturing followed steps enriching for melanized fungi and the addition of antibiotics to exclude bacteria (5). Growth of one black yeast strain was isolated and subcultured on MEYE at room temperature twice for axenic confirmation and designated TK_68. Genomic DNA was extracted from yeast colonies grown for 1 week on MEYE plates using a cetyltrimethylammonium bromide (CTAB) protocol (6). Genomic DNA was measured by Nanodrop and Qubit and diluted to ~28 ng/L. DNA sequencing libraries were prepared in the Institute for Integrative Genome Biology Genomics Core at University of California, Riverside (Riverside, CA) with Nextera DNA Flex Library kit and sequenced with 2 × 150 bp on Illumina NovoSeq 6000 in the QB3 Genomics Facility at the University of California, Berkeley.

**Fig 1:**
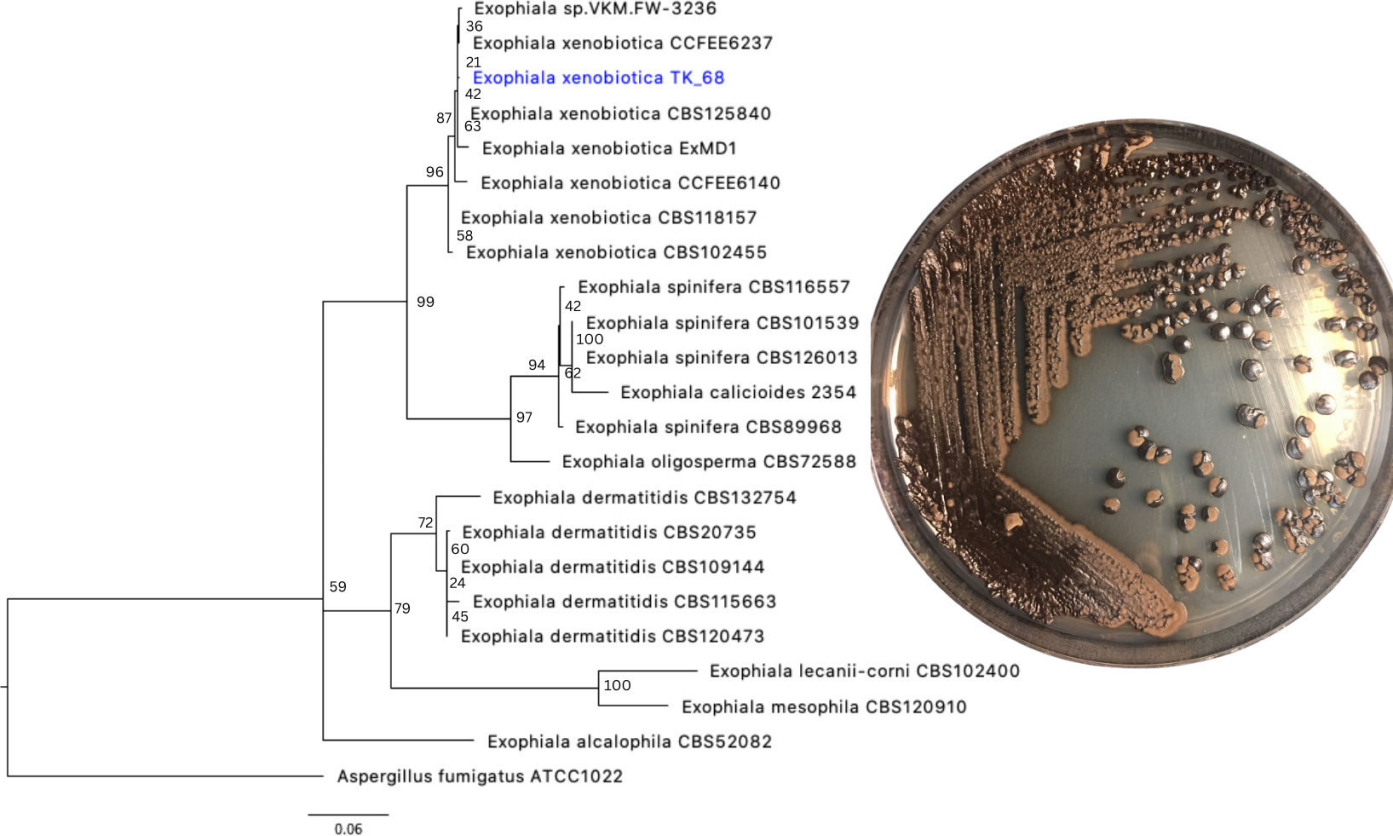
Agar culture of *Exophiala xenobiotica*, axenically isolated from the soil collected in box 5B-P23 C2A, and a phylogenetic tree describing our species along with respective 22 NCBI isolates. (**A**) Species identity was inferred from the phylogenetic tree constructed from ITS region sequences for our species from this study (in blue), 21 reference sequences from *Exophiala* genera, and rooted with one sequence of outgroup taxa.

The TK_68 strain was identified as (100% identity) *Exophiala xenobiotica*, accession number KX426972.1, by PCR amplifying the Internal Transcribed Spacer (ITS) region (28S + ITS1 + 5.8S + ITS2 + 18S) using ITS1F and ITS4 primers and amplification protocols (7), sequenced with Sanger sequencing, and BLASTN search to the NCBI refseq ITS database. To further confirm species identity, ITS regions were extracted with ITSx (*v.1.1.3*) (8) from public *Exophiala* genomes in GenBank and outgroup *Aspergillus fumigatus* ATCC 1022. A multiple sequence alignment of the ITS region was constructed with MUSCLE (*v.5.1*) (9), trimmed with ClipKit (*v.1.3.0*) (10), and the phylogenetic relationships inferred with IQTree2 (*v.2.2.2.6*) (11). The strain was deposited in the USDA-ARS NRRL collection as accession NRRL 64630 (Fig. 1).

The NRRL 64630 genome was assembled using the AAFTF pipeline (*v.0.2.3*) (12–16) performing filtering and trimming steps for data quality, relying on SPAdes (*3.15.4*) (17) for assembly, AAFTF tools for contaminant removal, and assembly polishing. Telomeres were calculated using the find_telomere.py script (18). BUSCO (*v.5.4.4*) ascomycota_odb10 database (19) was used to assess the completeness of the assembly. Genome annotation was performed with Funannotate (*v.1.8.10*) (20–38). Default parameters for the underlying tools were applied throughout. Genome sequencing, assembly, and protein-coding gene annotation statistics are summarized in Table 1.

**TABLE 1:** Strain and species designation, isolation source, sequencing read, assembly, and annotation statistics^*a*^

Species	Location	No. of read pairs	Coverage	No. of contigs	Genome size	Contig L_50_	Contig N_50_	G + C content (%)	Genome completion (BUSCO %)	Genome duplication (BUSCO %)	No. of genes	Telomeres found (forward, reverse, T2T)
Exophiala xenobiotica NRRL 64630	La Brea Tar Pits, Los Angeles, California	9,065,778	60.43×	682	30.28 Mb	15	676 kb	51.86	97.9%	0.0%	11,317	7 F 8 R, 0 T2T (both)

^*a*^ The species identification number for NRRL is listed, as is the location where the soil was collected from. The number of reads was used to help determine the coverage value. Genome assembly calculations include a number of contigs, genome size, N50, L50, and G + C content, while genome annotation results include a number of genes predicted and annotated. BUSCO completion statistics and comparisons were determined using the sordariomycetes_odb10 database with 3817 genes. Telomeres were calculated using the find_telomere.py script (Hiltunen et al. 2021).

## Data Availability

This Whole Genome project has been deposited at DDBJ/ENA/GenBank under the accession JAPDRM000000000.1 and SRA accession of SRR22028093. Genome assembly, annotation and phylogenetic assessment pipeline and related code are archived at DOI 10.5281/zenodo.8021900 (39). The culture has been deposited in the USDA ARS Culture Collection (NRRL) under the strain accession NRRL 64630.
